# You Get What You Pay for on Health Care Question and Answer Platforms: Nonparticipant Observational Study

**DOI:** 10.2196/13534

**Published:** 2020-01-15

**Authors:** Fatemeh Ameri, Kathleen Keeling, Reza Salehnejad

**Affiliations:** 1 Alliance Manchester Business School The University of Manchester Manchester United Kingdom

**Keywords:** internet health information, health information, health literacy, eHealth, information literacy, health care access

## Abstract

**Background:**

Seeking health information on the internet is very popular despite the debatable ability of lay users to evaluate the quality of health information and uneven quality of information available on the Web. Consulting the internet for health information is pervasive, particularly when other sources are inaccessible because of time, distance, and money constraints or when sensitive or embarrassing questions are to be explored. Question and answer (Q&A) platforms are Web-based services that provide personalized health advice upon the information seekers’ request. However, it is not clear how the quality of health advices is ensured on these platforms.

**Objective:**

The objective of this study was to identify how platform design impacts the quality of Web-based health advices and equal access to health information on the internet.

**Methods:**

A total of 900 Q&As were collected from 9 Q&A platforms with different design features. Data on the design features for each platform were generated. Paid physicians evaluated the data to quantify the quality of health advices. Guided by the literature, the design features that affected information quality were identified and recorded for each Q&A platform. The least absolute shrinkage and selection operator and unbiased regression tree methods were used for the analysis.

**Results:**

Q&A platform design and health advice quality were related. Expertise of information providers (beta=.48; *P*=.001), financial incentive (beta=.4; *P*=.001), external reputation (beta=.28; *P*=.002), and question quality (beta=.12; *P*=.001) best predicted health advice quality. Virtual incentive, Web 2.0 mechanisms, and reputation systems were not associated with health advice quality.

**Conclusions:**

Access to high-quality health advices on the internet is unequal and skewed toward high-income and high-literacy groups. However, there are possibilities to generate high-quality health advices for free.

## Introduction

### Background

Common drivers of the popular Web-based health care information seeking are serious health needs and inaccessibility of other sources in traditional settings [1] because of time, distance, and financial constraints or the value of a sense of control and empowerment or anonymity [2-5].

With resonance to the idea of *frugal innovations* in health care [6], so that “more can be done for less for many more people, globally” [7], the internet is seen as a low-cost and convenient way of accessing health information and care that could help in reducing the burden on health care systems. Bhatti et al recommend assessing affordability, adaptability, and accessibility [7]. For accessibility and adaptability, the pervasive adoption of mobile phones helps inclusion by providing wider opportunities for access. However, equal opportunity of access to the internet does not guarantee equal access to high-quality health information, which is variable across health topic areas [8]. Moreover, people find it difficult to find relevant information when they are using search engines to find answers for their question [9-11].

The quality of health information available on the Web has long been a matter of concern [12,13]. Several top-down initiatives have been developed to monitor quality, including MedPICS Certification and Rating of Trustworthy Health Information by Health on the Net Foundation (a European Union project for certification and rating of trustworthy and assessed health information on the internet) and Information Standard (an accreditation system established by UK health departments aimed at filtering unreliable Web-based health information). The success of such measures is bound to be limited. The rise of Web 2.0 (the second generation of the Web that is interactive and dynamic) has increased the complexity of Web-based health information provision [14,15]. The diversity of Web-based health information sources and the ongoing debate regarding Web-based health information quality question the effectiveness of top-down approaches.

Furthermore, accurate information can be taken out of context and cause harm. Users may concurrently pursue multiple medications following advices they receive on the Web, with adverse consequences. The use of search engines to locate relevant information can be challenging and requires experience [16]. Question and answer (Q&A) health care information platforms are Web-based services where information seekers may request personalized health advice. This can help alleviate the individual context or need issue, but the quality remains a matter of concern.

A threat to accessibility is that not all health care Q&A sites are free to use. The paradox is that advertisement-supported platforms where health information is free can often be perceived as less credible by users [17].

As a solution to the information quality issue, information management literature recognizes the relationship between platform design features and the quality of information generated on the platform. Q&A platforms embody technical and social choices that can impact user activities and the quality of Q&As [18], including eligibility constraints such as medical expertise for answering, tracking user contributions, reputation systems, revealing identities versus anonymity, and, crucially, cost of participation. It is unclear and under-researched whether these design features are effective in health domains. The common approach of relying on user feedback to rate information quality [19] is questionable in health domains, as lay users may lack the knowledge to evaluate health information [10,20]. Such concerns raise questions about the effect of platform design on health information quality in Q&A platforms.

Research on platform design features that promote the generation of high-quality advices is scarce in health care. This study reviews the literature to create a thorough list of design features that may impact information quality and examines their relationships with health advice quality on Q&A platforms. A unique contribution of this study is merging 3 levels of design features related to quality and examining their interactive effects on information quality in health Q&As. A further contribution is the research design and dataset. Using actual Q&As from Q&A websites and applying a nonparticipatory data collection method, where the observer does not participate in the social setting, improve the precision of the research results [21]. The research employs least absolute shrinkage and selection operator (LASSO) to deal with the initial large number of variables and uses unbiased regression tree to investigate possible interactions among design features. The approach offers valuable policy insights and recommendations for how to design Q&A platforms to maximize information quality.

### Model Development

Few studies focus on health information quality. We review major research streams on the effect of Web-based platform design features on information quality, classifying them into 3 categories: (1) firm level, (2) platform level, and (3) user level. Table 1 summarizes these research strands and defines the focus of this research. The main bodies of literature examined were information management, health services, designing Web-based platforms. The search began in Google scholar using the keywords “online health information quality.” From these results, the search was expanded by other keywords: “online information quality,” “information quality,” “answer quality,” “information sharing quality,” “knowledge sharing quality,” and “quality of online contribution.” Backward and forward search from highly relevant papers also expanded the search until it was determined that no new information was being uncovered. In the empirical section, we will test the relationship between these 3 levels of design features and health care advice quality.

**Table 1:** Model development summary and deficiencies filled by this study.

Level of analysis and factors			Studies	Focus of previous research	Focus of this study
**Firm level**					
	Revenue model (advertisement, transaction, membership fee)		Harper et al [22]; Enders et al [23]	Comparison of information quality in fee-based and free platforms; the role of revenue model in social networking sites	Effect of revenue model
**Platform level**					
	**Incentives**				
		Financial	Chen et al [24]; Harper et al [22]; Wang et al [25]; Hsieh et al [26]	Financial motivation for knowledge sharing	Effects of different types of financial incentives
		Nonfinancial	Chang and Chuang [27]; Chen et al [24]; Khansa et al [28]	Nonfinancial motivation for knowledge sharing, for example, altruism, social recognition, and social interaction	Effect of nonfinancial incentives in the form of points or credits
	Reputation (internal, external reputation)		Hung et al [29]; Chang and Chuang [27]; Tausczik and Pennebaker [30]	Comparing the effect of reputation and financial incentives; effect of external reputation	Effect of reputation
	Web 2.0 mechanisms		Khansa et al [28]	Effect of information technology–enabled incentives on knowledge sharing behavior	Effects of formal and informal mechanisms
**User level**					
	Users knowledge background (medical certification, expertise)		Reavley et al [31]; Giles [32]; Clauson et al [33]; Harper et al [22]; Oh [34]	Comparability of information quality provided by experts and crowd sourcing process	Effect of expertise
	Question quality		Hsieh and Counts [35]; Hsieh et al [26]	Variation of answer quality based on question type	Effect of question quality
**Across levels**					
	All the above factors		N/A^a^	N/A	The interaction and interplay of all design features

^a^N/A: not applicable.

### Firm-Level Design Features

Designing a revenue model is an essential part of platform design. Revenue can be generated from advertisements, membership fees, trading information, selling information to third parties, or a combination of any of these. Information economics theory suggests the revenue model choice may affect information quality. The key revenue driver for the advertisement-based revenue model is the number of users. However, maximizing Q&A numbers without regard to quality could lead to poor information generation [24,36]. Enders et al [23] argue that platforms earning revenue from information transactions attempt to maximize user willingness to pay by offering high-value information. From the user perspective, health information on advertisement-supported websites is often perceived as less credible [17], but this is moderated by context, such as whether websites might be expected or not to be allied to advertising [37]. The situation may be more nuanced than suggested by information economics.

### Platform-Level Design Features

Provision of quality information is costly. A rational participant will weigh the time and effort costs of providing information against its expected rewards. Adequate financial or social rewards are critical for ensuring provision of high-quality information [22,24,25,27]. An understanding of how different types of rewards, financial versus intrinsic, impact information quality is critical for equality of access to quality health information. For poor populations to benefit, the quality of health information on platforms where no fee is charged needs to be high.

#### Financial Incentive

Empirical studies on the effect of financial incentives on information quality present no clear results. For Google Answers (a platform using financial incentives), Chen et al found that higher financial incentives led to longer but not better answers [24], whereas Harper et al found Google Answers to have higher answer quality information compared with free Q&A sites (eg, Yahoo Answers and Live QnA) [22]. In an experimental study of product reviews, Wang et al [25] found no significant quality differences between reviews by paid and unpaid reviewers.

#### Nonfinancial Incentive

Several studies suggest the efficacy of nonfinancial incentives such as virtual points and credit to leverage motivations such as recognition or reputation building to encourage participation [24,27,29]. Participation is encouraged through reputation systems and virtual points or Web 2.0 mechanisms, such as voting or following. Oh and Syn [38] found different motivations associated with particular answering strategies. The association of these mechanisms to the quality of users’ contributions is under-researched.

Intrinsic motivations can motivate information providers to share their information. In social health Q&A, empathy with others who are going through similar pain and stress is a strong motivator. Oh [34] found altruism was the most influential factor for participation of health question answerers in social health Q&As.

A platform may encourage users to reveal their real identity or sign up using their social media account such Facebook or LinkedIn. This extends the reputation of the users to outside the platform and gives them higher incentives to co-operate, as they want to protect or promote their *external reputation* [30].

### User-Level Design Features

#### Expertise of the Information Provider

The role of information provider expertise is another contested question. For some, the quality of user-generated information in websites such as Wikipedia is comparable with, or even better than, the quality of information provided by experts in centrally controlled Web and printed sources [32]. In contrast, other studies found the opposite [31,33,39].

#### Quality of the Query

Shah et al [40] argue for a connection between an expressed information need and information quality provided. Indeed, Chen et al [24] indicate information providers are discouraged by insufficient numbers of high-quality questions on an information platform. If information seekers post nonserious or trivial questions, or are allowed to post unrelated issues, they automatically waste valuable time and the attention of the potential answerers [35]. Relatedly, information seekers can themselves increase answer information quality by increasing question quality [22].

### Across Levels: Combination of Design Features

Each Web-based information platform contains a mixed set of design features. It is unlikely that a single design feature uniquely shapes information quality. From both a theoretical and practical perspective, it is critical to investigate the complementary role of design features. There is some evidence of such interactions for select website features [26], but to the best of our knowledge, there is no research investigating design features associated with quality at a comprehensive level.

## Methods

### Research Design

On July 1, 2014, we used 4 search engines (Google [41], Yahoo! [42], Bing [43], and Ask.com [44]) to search the terms “question and answer sites or platform or website.” Our search was in English but not confined to any particular country. We chose the top 200 search results and obtained a total of 40 Q&A platforms. We excluded the nonhealth Q&A platforms and analyzed them to identify a set whose design features contained the variation of design features described above. In the case of similarity of mechanisms of platforms, popularity of the platform both among the internet users and in the literature made a platform more favorable for selection. This resulted in the choice of 9 platforms: AllExperts, AnswerBag, ChaCha, Google Answers, JustAnswer, Mahalo Answers, Quora, WebMD, and Yahoo Answers. Although Google Answers and Mahalo are no longer active, they are included in our sample because they represent design features not embodied in active platforms (see Multimedia Appendix 1 for description of the design features of the nominated platforms). One of the research aims was looking at how different incentives and user knowledge might affect the quality of answers; therefore, the unit of analysis was *1 question and 1 answer*, which corresponded with the analytic strategy of this exploratory study. In cases where a conversation took place between a single questioner and responder, the whole thread was evaluated. In addition, *1 question and 1 answer* were consistent with platforms that do not allow more than 1 answer.

Recruiting human raters to assess information quality is a common practice in the literature [24,26,45]. We recruited National Health Service–certified physicians and trained them (July 2014) to increase the validity and reliability of the quality evaluation. Following the random selection of 100 Q&As from each Q&A platform over the period of 6 weeks (900 in total) in July and August 2014, 2 physicians conducted the evaluations, each of them rated half of the data plus 10% to allow an inter-rater reliability check (see Multimedia Appendix 2 for details of the data selection and rating process). A first layer of anonymity was provided by the coders being *blind* to the name of the Q&A website where the question was asked and any other attributes associated to the Q&As. There is little risk to individual anonymity, as this level of detail is removed during the coding process. The research was conducted under the research ethics rules and policies of the University of Manchester, United Kingdom.

### Dependent Variables

Many attempts have been made to identify the criteria to evaluate Web-based health information [15,46]. Included in these is the extensive literature review by Oh et al [47] that developed the following criteria for measuring health advice quality in Q&A platforms: accuracy, completeness, relevance, objectivity, readability, and source credibility. See Table 2 for definitions. These criteria formed the basis for our research based on the relevance to the study [47] and from medical point of view.

The 2 assessors rated the answers on each measure (5-point Likert scale, with 5=very high and 1=very low quality). We conducted principal component analysis (PCA) to make an index of answer quality from the primary measures, and 1 factor with an eigenvalue >1.0 was extracted from the PCA (see Multimedia Appendix 3 for PCA details). Thus, we built a single composite measure by averaging the sum of the individual criteria ratings.

**Table 2:** Health answer quality measures and definitions.

Criteria	Explanation
Accuracy	The answer provides correct information, that is, degree of concordance of the information provided with the best evidence or with generally accepted medical practice
Completeness	The answer includes all key points
Relevance	The answer is relevant to the question
Objectivity	The answer provides objective and unbiased information, for example, addresses all considerations of an issue, judgement does not appear to be swayed by considerations of self-interest or prejudice
Readability	The answer is easily readable, for example, organized, simple language, explanation of medical terms, and shorter sentences and paragraphs
Source credibility	The source of information is authoritative, for example, capable of being verified, does not seem to have commercial intent or personal agenda. Not applicable when no source is provided

As a check of the inter-rater reliability between 2 assessors, they independently rated the information quality of a common 10% (90 Q&As) of the data. We report inter-rater agreement measures for variables in the form that was used for model testing (ie, average indices) rather than the raw form [48,49]. We analyzed intraclass correlation coefficient (ICC) values using a single measurement, absolute agreement, 2-way random effects model. The ICC values for question quality index was 0.721 and for answer quality index was 0.764 with 95% confidence interval. Both quality indices exceeded 0.7, which indicates medium-to-good reliability [50].

### Independent Variables

The independent variables are question quality and the platform design features.

To evaluate question quality, we used the criteria proposed by Harper et al [51] and Hsieh et al [26]: importance, perceived urgency, difficulty, question archival value, and writing quality. See Table 3 for definitions. PCA reveals only 1 eigenvalue >1.0 extracted from these measures of question quality (see Multimedia Appendix 3). Therefore, we built a single question quality composite measure by averaging the sum of the individual criteria ratings.

Each of the 9 selected Q&A platforms was reviewed by the first author to identify and record the presence of platform design features previously identified: incentives, Web 2.0 mechanisms, revenue models, expertise of participants, and question quality (see Table 4). LASSO allows high flexibility for inclusion of design features, as it does not impose constraints on the number of independent variables.

**Table 3:** Health question quality measures and definitions.

Quality criteria	Explanation
Importance	How seriously/sincerely did the questioner want an answer to the question? (eg, absence of reason for posting other than seeking information, eg, self-promotion/advertising product)
Perceived urgency	How urgently did the questioner want an answer to the question?
Difficulty	How difficult is the question to answer? (low and very low—anybody can answer the question; neither high nor low—an average high school–educated person is able to answer the question; high—someone with general medical background can answer the question; very high—specialist can answer the question)
Question archival value	Answer to this question will provide appropriate and adequate coverage of the issue to provide information of lasting/archival value to others
Writing quality	The question is well written (clear question, focused, and summarizes the issue)

**Table 4:** Variable descriptions.

Category and variables			Explanations
**Firm level**			
		Advertisement-based revenue; transaction-based revenue	Platform can use advertising or transaction-based model, both models, or neither model
**Platform level**			
	**Financial incentives**		
		Financial interest	Whether the information provider has any type of financial interest, either actually getting paid or with prospect of getting paid in future. In some cases, the advice provider was not actually paid but had the prospect of being hired by the platforms in the future
		Payment for answering	Whether or not the information provider has been paid?
		Variable payment scheme	The financial incentive could be paid in fixed or variable rate determined between the advice provider and asker
		Amount of payment	How much money has been paid to information provider?
	**Nonfinancial incentives**		
		Virtual incentives	Whether any type of nonfinancial incentive, such as virtual points and credits, was involved?
		Internal reputation system	Internal reputation system maintains and publicizes users’ activity within a platform and their profiles
		External reputation	External reputation is the identity and reputation of the users outside the platform in Web- and non-Web-based worlds
	**Mechanisms**		
		Web 2.0 mechanisms (voting, following)	These mechanisms reflect the feedback of users on each other’s activity
**User level**			
	Expert		The expertise of participants refers to their medical certification or their researching skill that is certified by the platform
	Question quality		The quality of raised question

### Analytical Methods

Our sample formed a wide dataset in that it included a large number of predictors with a comparatively small number of observations on the predictors. This feature of the sample makes it practically impossible to apply popular ordinary least square (OLS) regression techniques to model the data. A limitation of OLS regression for wide data concerns overfitting bias. Applying OLS to wide data can give rise to higher variance and poor out-of-sample predictions. LASSO provides suitable alternatives for modeling wide data [52]. It reveals and calculates the coefficients of predictively significant variables that minimize out-of-sample prediction error [53].

**Figure 1:**
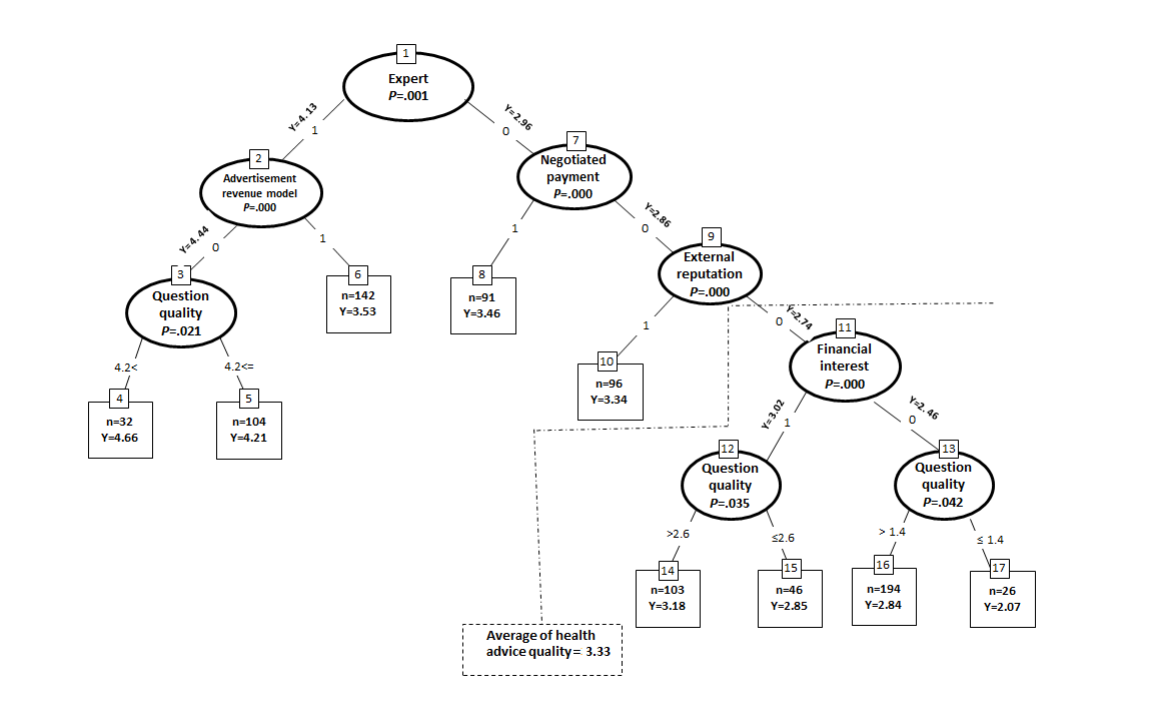
The unbiased regression tree (N=834). "n" is the number of records; "Y" is the value of health advice quality.

To provide a more nuanced explanation by identifying the interactions among predictors, we created an unbiased regression tree to study interactions among predictors (see Figure 1). The technique involves segmenting the predictor space into several regions. To make a prediction for a given observation, the method typically uses the mean of the training observations in the region to which it belongs. The splitting rules used to segment the predictor space are summarized in a tree structure. The advantage of regression tree is that it reveals conditional relationships, that is, how multiple combinations of design features are related to health advice quality, as it does not fit a linear model to the entire response space, it identifies conditional relationships between predictors and the response, and it presents the results in the form of a tree. Predictors appearing in higher layers of the tree or multiple times are predictively more significant than variables occurring in lower layers. Given the initial set of variables entering the tree, the variables absent from the tree do not improve prediction accuracy [54] (for analytical methods see Multimedia Appendix 4). To the best of our knowledge, this is one of very few studies that exploit LASSO and unbiased regression tree to determine design features that affect health advice quality.

## Results

### Least Absolute Shrinkage and Selection Operator

The composite measure of question answer quality had a mean of 3.33, standard deviation of 1.24, and median of 3.6 (N=834). The question quality composite measure had a mean of 3.28, standard deviation of 0.81, and median of 3.6 (N=900). We found that variables representing expertise of information providers, payment for answering questions, external reputation, revenue model, and question quality were the best predictors of health advice quality. The variable representing the use of an advertisement-based revenue model had negative association with health advice quality. Variables measuring nonfinancial incentives, internal reputation systems, and Web 2.0 mechanisms were not significant predictors. Table 5 presents LASSO coefficients, standard errors, and *P* values.

The initial analysis using LASSO explains the primary linear relationship between the best predictors and the outcome variable, health advice quality. The R-squared of the model was equal to 26%, which confirmed that the design of a Q&A platform could explain part of the advice quality. This is the basis for an overall understanding of the relationships between design features and health advice quality. However, at a more nuanced level of understanding, there may be interactive relationships between the predictors and the outcome that are apparent under certain conditions that can be explored using nonparametric techniques, such as regression tree, to extract the conditional relationships between design features and health advice quality.

**Table 5:** Least absolute shrinkage and selection operator results for the dependent variables.

Category and dependent variable			Regularization parameter		LASSO^a^ coefficients		SE		*P* value	
Intercept			2.24		—^b^		—		—	
**Firm level**										
	Advertisement-based revenue			−0.27		−0.25		0.07		<.001
	Transaction-based revenue			0.11		0.26		0.09		.01
**Platform level**										
	**Financial incentives**					0.40		0.08		<.001
		Financial interest		0.00						
		Payment for answering		0.25						
		Negotiated payment		0.00						
		Amount of payment		0.00						
	**Nonfinancial incentives**					—		—		—
		Virtual incentives		0.00						
	**Reputation**					0.29		0.09		<.001
		Internal		0.00						
		External		0.10						
	Web 2.0 mechanisms (voting, following)			0.00		—		—		—
**User level**										
	Certify			0.00		—		—		—
	Expert			0.59		0.48		0.07		<.001
	Question quality			0.06		0.12		0.03		<.001
Number of records			834		—		—		—	
Root-mean-square error			0.82		—		—		—	
R-squared			0.26		—		—		—	

^a^LASSO: least absolute shrinkage and selection operator.

^b^Not applicable.

### Unbiased Regression Tree

The result of the regression tree is summarized in Figure 1 and shows that expertise was the most significant factor in predicting health advice quality because it appeared at the root node, supporting the LASSO results. In presence of experts, the second layer (node 2) distinguished between platforms where experts responded to health queries and advertising played no role and those where experts responded but advertising was a part of the revenue model. In the former case, there was further differentiation by question quality: when the mean question quality exceeded 4.2 (node 4), the mean answer quality was the highest (4.66). Question quality ≤4.2 (node 5) was associated with lower-quality health advices. Where advertising plays a role (node 6), answer quality is lower, that is, lower-quality answers were associated with platforms where no financial incentive was offered and providing information was on voluntary basis for philanthropic purposes of helping others or building external reputation. The interesting point is that, even so, the quality rating was above average for the dataset. So, although the advertisement-based revenue model had a negative relationship with quality, the strong effect of experts could alleviate this effect, and above-average quality was produced.

The other side of the tree concerns the absence of experts to provide answers. In the absence of experts, whether the advice provider receives any financial rewards and the platform allows the asker and answerer to directly agree on a price rather than establishing a fixed price per question (node 7) appeared as the second most important factor, and advice quality was still above average for the dataset (node 8). In node 9 where experts and variable payment scheme were absent, external reputation was associated with higher than average advice quality. In the next level where external reputation was absent (node 11), financial interest showed up. It means that advice providers with the prospect of getting paid in the future generated higher quality health advices compared with those who have no financial interest. Here, higher-quality questions were associated with higher-quality heath advices (node 14), although the quality is now below average for the dataset. The lowest quality health advice was generated in the absence of experts, motivation of external reputation, and any type of financial interest where low-quality information was raised (node 17).

## Discussion

### Overview

This study investigates the complexity of the relationship between design features of Web-based platforms and the quality of health advice generated in them. Theoretical and empirical evidence suggest a wide range of potential design features at different levels associated with health advice quality. The LASSO selects the design features that best predict the quality, and the regression tree identifies interactions among the design features.

### Firm-Level Features

#### Revenue Model

Higher health advice quality is associated with the transaction-based revenue model, whereas lower advice quality is associated with an advertisement-based revenue model. This finding reinforces the findings of Harper et al [22], suggesting answer quality is superior in fee-based Web-based Q&A platforms than free sites. Taken on its own, this has worrying implications for these platforms to be instrumental in equality of Web access to quality health care.

Notwithstanding this intuitive result, the results also suggest that external reputation, expertise of the answerer, and question quality are also associated with higher quality. The revenue model is negatively associated in the LASSO regression models, which is in line with the worries regarding the advertisement-based revenue model, that is, these platforms tend to maximize the number of visitors at the expense of advice quality. On such platforms, no restrictions are placed on question quality or subject of postings; neither are users’ efforts for creating high-quality advice recognized nor compensated. Although this is a common issue in all types of platforms, the potential consequences of poor advice, for example, incorrect diagnosis, users employing incorrect treatments, or ignoring correct treatments, are more serious in the health domain.

Nonetheless, as suggested in the introduction, the situation is more nuanced. The regression tree analysis refines our view about the advertisement-based model; we find indications of answer quality on these platforms that exceeds the average when experts give answers. In other words, the effect of experts in the health domain is so strong that the negative effect of the advertisement-based revenue model is controlled.

### Platform Level

The results reveal that financial incentives are very influential in forming quality of health advice in different circumstances. First, there is a linear relationship between *payment for answering* and quality of health advice. Second, paid experts are providing higher quality than unpaid experts. Third, a variable payment scheme is associated with above-average quality advice. Finally, lay users with the prospect of getting paid in the feature are providing higher-quality answers comparing with those who have no financial interest at all.

Kissick [55] argues at a general level that quality, cost, and access are 3 essential, but competing, features of health care systems. This study confirms this argument in accessing Web-based health advice. High-quality advice is primarily accessible for payment; thus, access is compromised.

Web-based platforms such as eBay, TripAdvisor, and Amazon ask current users of their products/services to evaluate the quality of them after consumption. This feedback is aggregated and enables more informed decisions of future users. However, the usefulness of such mechanisms is questionable in health domain because of the nature of health advice as a *credence good*. Users can evaluate the quality of experience goods such as clothing, furniture, restaurant, hotel, etc. However, the quality of credence goods such as health care advice is difficult to evaluate for lay users even after consumption because they lack a medical background [56]. This undermines the effectiveness of any mechanism that works based on users’ feedback in the health domain. Our results confirm that there is little association between these mechanisms, for example, Web 2.0 mechanisms and reputation systems and an external, independent assessment of health advice quality.

On the Web-based health platforms that rely purely on such mechanisms, this issue can result in the situation where poor-quality crowds out higher quality [57]. This means that in absence of a system that signals quality, contributing high-quality advice is not recognized. This leaves no motivation for advice providers to share quality advice, resulting in the platform comprising low-quality contributions.

Similarly, there is no evidence for effectiveness of virtual incentives. This supports the theory indicating that repeated positive rewards lose their value over time [58]. Therefore, reputation management may not need to be highly emphasized on Web-based information platforms because it may engage people at the early stage of being a platform user but be of value for long-term users, especially advanced answerers [28,38].

However, the results strongly suggest that above-average health advice quality by nonexperts can be produced when external reputation is used on the platform.

### User Level

#### Expertise of Respondents

Crowd sourcing platforms rely on small contributions by a large number of people being superior to the contributions of a few experts [59]. However, our analyses suggest that in health care, advice provider expertise is the most effective predictor of answer quality. Experts, whether incentivized by financial or social rewards, are associated with the highest-quality answers.

It is surprising to see that volunteer health experts provide lower-quality health advice comparing with those who are paid. This might be explained by the lack of face-to-face interaction on Web-based platforms, and thus, lack of feedback on helpfulness of the contribution to receivers that decreases the satisfaction they get for helping others.

#### Question Quality

Altruism and enjoyment are among the top-ranked motivations of participation [34,38]. High question quality means clearer questions. Therefore, information providers have higher motivation to provide higher-quality answers to high-quality questions. Our analyses support this view as high-quality answers are associated with high-quality questions, consistent with Hsieh et al [35] and Harper et al [51].

### Across Levels

Regression tree analysis results show that a combination of experts, financial incentives, and high-quality questions predicts the highest health advice quality, suggesting that the highest quality is produced when experts are motivated by a combination of personal gain in the form of financial incentives, high enjoyment, and altruism provoked by high-quality questions. Recruiting unpaid experts, incentivizing lay users financially while using variable payment schemes, and incorporating external reputation are all associated with higher than average health care advice quality. Lowest quality advice occurs where there are no experts, any form of financial incentive is absent, and question quality is poor.

The tree analysis provides further insights. First, even paid experts provide higher-quality answers to higher-quality queries. Second, if an advertisement-based revenue platform can recruit expert respondents, this is related to answer quality beyond the average. In the absence of financial incentives and experts, external reputation is the best predictor of advice quality. Finally, the question quality has an effect, even on platforms where the clusters of features associated with poor quality are present.

### Implications

The main contribution of this study is providing insights on the multiple relationships between the platform design and health care advice quality, and thus on Web-based health information platform design. Therefore, it has implications for health policy makers who attempt to address quality concerns and those concerned with access to Web-based health care information as a means for doing more for less for more people.

An important policy concern is inequality of access to quality advice on the internet. The findings show that the highest-quality advice is accessible for payment; it is not available for free, although only 2% of those seeking health information pay for it [60]. The connection between question quality and answer quality increases the danger of a triple hazard for low-income lower-literacy groups because of their lower ability to produce a quality question combined with a lower understanding of what is likely to be a lower-quality answer. Groups with low income and/or lower literacy are disadvantaged in accessing high-quality advice, increasing risks of electronic health (eHealth) inequality.

However, there is a potential to provide the highest-quality health advice for free, if the platform pays the recruited experts but does not charge the consumers. YouTube provides free access to all its videos but pays a fraction of its revenue from advertisements to video creators. Similarly, if the health platform pays experts from alternative revenue sources such as advertisement, selling data, and government subsidization, the highest quality can be generated and equally accessible for free.

In addition, designers can also consider the needs of the less well-educated, with low literacy or language barriers, for example, by providing voice or translation facilities, as Chu et al [61] recommended for other eHealth platforms.

The findings cast doubts on the effectiveness of common *internal* quality assessment features, such as voting and reputation systems, in the health domain and suggest using external reputation of the advice providers outside the platform.

Despite evidence that users form their own perceptions of information quality, platforms with objectively low advice quality are still available and viable, suggesting they answer some user needs, perhaps less available elsewhere. Lower question/answer quality is associated with less restrictions on asking or answering questions, these may go *off topic* and provide social support from fellow sufferers not found in more formal platforms, and the sheer volume of users may assist in assuaging feelings of facing an illness alone. Huxley et al [62] also report that marginalized groups find *stigmatizing reactions* a barrier. This may occur in more formal platforms, however, inadvertently. Studies of eHealth inequalities find that low literacy, low education, and other language difficulties are barriers to health information use [63]. Where answers are not provided by experts, the language may be less formal and more easily understood.

### Limitations and Future Research

Our results show 26% explained variance. However, in a study of this kind with noisy, high-variability data where R-squared values are likely to be low, we found the explained variance for the emergent features was significantly different from 0, indicating a statistically significant explanatory power. Hence, we believe our results show significant patterns that provide valuable information of practical significance from which important conclusions can be drawn. Nonetheless, our sample is limited in the sense that it only captures, perhaps partially, design features of the existing platforms. There are a host of other factors that are likely to affect information quality. An example is the platform popularity. It might be that higher-quality information providers are more likely to join popular platforms. The reputation of the platform owner such as Yahoo! might also impact the decision to join a platform. Although we did not have access to such data, including such factors might further explain the variation in quality. The text also mentions the case of donation- or charity-based Q&A platforms. Recent advances in text recognition have also paved the way for effective information aggregation and comparing the quality of an advice with past answers to similar questions. Although we faced challenges in including such factors, they are likely to add to the explanatory power of the model. The choice of design features was also limited to existing platforms. There might be alternative designs that better facilitate generation of high-quality information. An example is given in the text: donation- or charity-based revenue models might lead to higher-quality answers and extend access. In general, as in other platforms, an important avenue for learning about better design is experimentation. Future research should conjecture new designs and conduct A/B testing to learn about the effectiveness of the design features. Major platforms such as Amazon, Alphabet, eBay, and Facebook have turned to experimentation to improve their platforms. This can equally benefit the design of health information platforms. Advances in artificial intelligence and text mining techniques are bound to impact health information platforms. These advances will raise challenges for future research. Methodologically, future research can use recently developed causal regression trees to identify design features that drive information quality. This research rated health advice quality from medical point of view; future research should investigate the user perspective concerning health advice quality, especially in view of potential barriers for disadvantaged or marginalized groups. Further research is needed to explain why volunteer unpaid health experts and paid experts provide uneven health quality advice in the Web-based world. Furthermore, we considered only English language platforms, leaving scope for future cross-cultural investigation. We used a robust search strategy to identify the design features; however, the inclusion of design feature was based on judgement of one coder, and in addition, we did not adopt *systematic search*.

## Appendix

Multimedia Appendix 1 Platforms.

Multimedia Appendix 2 Rating process.

Multimedia Appendix 3 Principal component analysis.

Multimedia Appendix 4 Analytical methods.

## Abbreviations

eHealth: electronic health
ICC: intraclass correlation coefficient
LASSO: least absolute shrinkage and selection operator
OLS: ordinary least square
PCA: principal component analysis
Q&A: question and answer
