# Supplementary material for: You Get What You Pay for on Health Care Question and Answer Platforms: Nonparticipant Observational Study
Source: J Med Internet Res. 2020 Jan 15;22(1):e13534. doi: 10.2196/13534 (PMC6996747; doi:10.2196/13534)
Supplement: Multimedia Appendix 1 [file jmir_v22i1e13534_app1.docx]

# Appendix 1

# Table1: of reviewed platforms

| **Platform Name** | **Web address** | **Why not chosen?** |
| --- | --- | --- |
| 1. **Able2Know** | <http://www.able2know.org> | Access to data |
| 1. **AllExperts** | <http://www.allexperts.com> | Chosen |
| 1. **Answer.com** | <http://answers.ask.com> | Answers are generated partly by scraping and crawling |
| 1. **Answerbag** | <http://www.answerbag.com> | Chosen |
| 1. **AOL Answer** | <http://aolanswers.com> | Similarity to Yahoo Answers (40) and Answerbag (4) |
| 1. **Ask a librarian** | <http://www.askalibrarian.org> | Focuses on education |
| 1. **Ask Me Help Desk** | <https://www.askmehelpdesk.com> | Similarity to 4 and 40 |
| 1. **Ask Meta Filter** | <http://ask.metafilter.com> | Contains community features rather than Q&A platform |
| 1. **Ask The Answer** | <http://www.asktheanswer.com> | Focuses on entertainment |
| 1. **Askville** | <http://askville.amazon.com> | Similarity to 4 and 40 |
| 1. **Baidu Knows** | <http://zhidao.baidu.com> | Chinese |
| 1. **Blurt it** | <http://www.blurtit.com> | Chosen |
| 1. **ChaCha** | <http://www.chacha.com> | Chosen |
| 1. **Cramster** | <http://www.cramster.com> | Focuses on education |
| 1. **Dizzay** | http:// www.dizzay.com | Access to data |
| 1. **Expert Exchange** | <http://www.experts-exchange.com> | Focuses on technology |
| 1. **Fluther.com** | <http://www.fluther.com> | Similarity to 4 and 40 |
| 1. **Girls ask Guys** | http://www.girlsaskguys.com | Focuses on relationships |
| 1. **Google Answers** | <http://answers.google.com> | Chosen |
| 1. **Helpfulbox** | <http://myhelptopicsforum.com> | Similarity to 4 and 40 |
| 1. **Just Answer** | <http://www.justanswer.com> | Chosen |
| 1. **Just Ask** | <http://www.education.com/answers> | It focuses on education |
| 1. **KGB** | www.kgbanswers.com | Data access |
| 1. **Ask From Expert** | <http://www.askfromexpert.com/> | Similar to Just Answer |
| 1. **Knowledge-iN** | <http://kin.naver.com> | Korean |
| 1. **Linked In Answer** | <http://www.linkedin.com/answers> | It is closed |
| 1. **Mahalo Answers** | www.mahalo.com/answers | Chosen |
| 1. **Marchant Circle Answers** | <http://www.merchantcircle.com/answers> | It focuses on business |
| 1. **Minti question and answer** | <http://www.Minti.com> | It focuses on parenting |
| 1. **Questions** | <https://www.question.com> | Similar to 4 and 40 |
| 1. **Quora** | <http://www.quora.com> | Chosen |
| 1. **Stackoverflow** | <http://stackoverflow.com/> | It focuses on technology |
| 1. **The Answer Bank** | <http://www.theanswerbank.co.uk> | It is a general purpose Q&A platform, no health category |
| 1. **True Knowledge** | <http://www.evi.com/> | Answers produced through an automated procedure and not by a human |
| 1. **Trulia** | <http://www.trulia.com/voices/> | Real state Q&A platform |
| 1. **Uclue** | <http://www.Uclue.com> | Similar to Google Answers but less popular |
| 1. **WebMD** | <http://www.webmd.com/> | Chosen |
| 1. **Wikia** | <http://wiki.answers.com/> | Similar to 4 and 40 |
| 1. **WiseGeek** | <http://www.wisegeek.com> | It is an archive of answers for common questions |
| 1. **Yahoo Answers** | <http://answers.yahoo.com> | Chosen |

# Description of the nominated Q&A platforms

This section elaborates the dynamism of asking and answering questions on the nine nominated websites and explains how quality is managed by them. The information in this section has been gathered from multiple sources. The main source was the platform itself and guidelines available for users. The authors also created an account and joined the community and raised at least three question in each platform to understand how asking and answering is occurring there. In the case of ambiguity the asking service of the website was used to get an answer from the community members. Furthermore, the platforms were contacted directly to verify the reliability of information. Community blogs such as yanswersblog.com, which is a blog for Yahoo Answers members, were also examined as another source of information.

#### Google Answers

Google Answers is a transaction-based information market launched by Google in 2002. Although the service was closed in late 2006, archived questions and answers are still accessible online. According to the online archive (http://answers.google.com/answers/) 53087 questions were asked across 10 categories^^[[1]](#footnote-1)^^, out of which 2398 were under the health category. Users could post a question and specify how much they were willing to pay for an answer, from $2 to $200. Google retained 25% of the researcher’s reward and a 50 cent listing fee per question. A client who was not satisfied for whatever reason could receive a refund less the listing fee. However, a satisfied client could leave a tip of up to $100. Answers were provided by Google Answers Researchers who were experts at locating hard-to-find information on the web. Researchers were required to go through an application process that tested their research skills and the quality of their answers. However, there was no claim for subject expertise of the researchers (Y. Chen et al., 2010). In addition to researchers, non-researchers could comment on the questions for free. Google claimed researchers could be recruited from commenters (Y. Chen et al., 2010).

Google Answers utilised a reputation mechanism to increase the quality of researchers’ contributions. Right after receiving an answer, clients were asked to rate the answer on a 1 to 5 stars. These ratings were averaged and shown as a part of the researcher’s reputation. The following information was available about the researchers: (1) ‘Average answer rating’ (1 to 5 stars): Right after receiving an answer, clients were asked to rate the quality of the answer on a one- to five-star system; ( 2) ‘Questions answered’: Total number of questions answered by the researcher; (3) ‘Total number of refunds’: Google Answers guaranteed money back in case of dissatisfaction by the provided answer; (4) All the questions answered by the researcher along with their respective ratings.

#### Yahoo Answers

Yahoo Answers is a social question and answer platform offered by Yahoo. It is recognised as the most widely used social question and answer platform on which more than 300 million questions have been posted in seven years since its launch in 2005 (yanswersblog.com). Registered users can post their question in a wide range of 26 categories^^[[2]](#footnote-2)^^ (including health) defined by Yahoo Answers and get an answer from fellow users. Posting questions and answers is free of any financial charge and Yahoo Answers generates its revenue out of advertisement. Yahoo Answers has a system of points and levels to manage participation of its users. Once a user begins participating on Yahoo Answers, he gets 100 points. Asking a question costs 5 points and providing an answer earns 2 points. A question may receive several answers and the original asker has the right to pick ‘The best answer’ among them which gives 3 points to the asker and 10 point to the answerer (for a complete list of points and levels see Appendix C4). If the asker does not pick the best answer in a particular time, the community votes for picking the best answer. Any user can express their opinion about an answer by either commenting or casting thumbs up or thumbs down.

In order to allow everyone to recognise how active and helpful a user has been in Yahoo Answers, the following information is available on the profile of every user: (1) Points: They are calculated based on users’ activities and cannot be used to buy or redeem anything; (2) Best answers: it represents the percentage of best answers provided by the user; (3) Answers: Number of answers; (4) Questions: Number of questions; (5) The user may decide to make all the questions and answers she provided publicly available. Leader board is another mechanism to encourage participation. It ranks and advertises the top contributors based on their accumulated points.

#### AllExperts

AllExperts is an expert question and answer platform founded in early 1998. It claims that all of its experts are volunteers with knowledge in their area of expertise. In order to volunteer, experts need to apply and state their educational credentials, organisations to which they belong, publications, etc. Accepted applicants are allowed to answer questions in AllExperts. As AllExperts advertises the incentive of volunteering is ‘getting traffic and attention’^^[[3]](#footnote-3)^^ and helping others; however, the revenue model is based on advertisement. To ask a question, users must first find an appropriate “expert” by navigating through a taxonomy of a wide range of categories provided by AllExperts. Questions will become publicly available if the asker allows.

Users can look at experts’ personal profiles and the ratings of their past answers before asking their questions. The reputation system on AllExperts uses the following aspects: (1) Knowledgeable, Clarity of Response, Timeliness and Politeness: these ratings are given in the interval [1, 10]. The score in each aspect is simply the numerical average of ratings received; (2) the number of questions an expert has received; (3) prestige score: volunteers get 30 prestige points every time they receive a knowledge rating over 7; (4) all the questions answered by the expert along with their respective ratings.

#### Just Answer

Just Answer is an online expert [question and answer](http://en.wikipedia.org/wiki/Knowledge_market) [platform](http://en.wikipedia.org/wiki/Website) launched in 2003. It provides answers in several categories^^[[4]](#footnote-4)^^ including Health & Medical. All experts must complete an application and have their credentials verified to be able to answer questions. A question costs **£**11 to **£**48 based on ‘urgency’ and ‘level of detail’ required for the answer and in case of dissatisfaction with the answer money back is guaranteed, however **£**5 is charged and retained by Just Answer upon posting a question. New experts earn 25% of what a customer is offering for an answer and this amount goes up to 50% as the experts get more experienced.

Upon receiving an answer, the customers are asked to rate the expert in 5 rating options: a score of one and two shows dissatisfaction and refund request while providing a rating of three (OK service), four (good service) or five (excellent service) authorises payment to the experts. The following information is available about the experts on their profile: number of satisfied customers; number of excellent services, good services and OK services provided by the expert in 3 months, in 12 months and in a lifetime period; in addition to the answers they provided.

#### Answerbag

Answerbag is a social question and answer platform where questions are asked and answered by users about any topic including health. Similar to Yahoo Answers, posting questions and answers is free of financial charge and the revenue model is based on advertisement. Unlike Yahoo Answers’ point system, users do not lose or earn any points for simply asking and answering; rather, earning points is for submitting good questions and answers. For instance: receiving likes on a question or answer yields 1 point, and if an answer is marked as ‘great’ by staff, moderators or community leaders, the answerer receives 5 points. Reporting in appropriate content is encouraged in Answerbag by a flagging mechanism. Flagged questions/answers are reviewed by moderators, and if they agree with the flag, they will give the user who flagged the question/answer 5 points.

Users have profile pages where their participation statistics are posted, including the categories in which they post answers, along with points and levels, and a list of their friends.

Users can rate both questions and answers by giving positive or negative points, from plus or minus 1 for beginners, to plus or minus 6 for very experienced users. Through their contributions to the site, users can “level up,” and earn the right to give or take away more points from other users’ questions and answers. Users can also accumulate points by flagging questions and answers as “Wrong Category”, “Nonsense”, “Spam/Offensive”, and “Duplicate”. Flagged questions/answers are reviewed by moderators, and if they agree with the flag, they will give the user who flagged the question/answer 5 points. Users have profile pages where their points and submissions are reviewable by other users.

#### ChaCha

ChaCha is a free and mainly mobile-based question and answer platform in the US launched in 2006. Users can send their question via text messaging^^[[5]](#footnote-5)^^, online or using mobile applications. Asking questions in ChaCha is free and questions are provided by guides who earn $0.02 per completed transaction. The ChaCha revenue model is based on advertisement while it pays a cut of its earning to guides to provide high-quality, accurate answers. A ChaCha guide applicant must complete a two-hour evaluation assessing how quickly and efficiently one can search the Internet for answers to questions (Bliss, Lodyga, Bochantin, & Null, 2010); however, no information is publicly available about the reputation of the guides.

#### Mahalo Answers

Mahalo launched a question and answer service called Mahalo Answers in late 2008 which was discontinued for no announced reason in 2013. Mahalo Answers allowed users to post questions regarding a wide variety of subjects including health, and those questions could be answered by fellow users. Similar to Yahoo Answers and Answerbag it used a point and level system. For instance, answering a question earned 2 points while posting a question cost no points. A key distinction was allowing questioners to give a monetary reward in Mahalo Dollars to the user who provides the best answer. The original asker had the right to select the best answer or choose ”no best answer” within four days, then the community voted for the best answer. Once answerers had earned more than 40 Mahalo Dollars, they could choose to cash out and Mahalo Answers took a 25% cut (10 Mahalo Dollar was redeemable to 1 US Dollar). Advertisement was another source of revenue for Mahalo Answers. One key difference between Google Answers and Mahalo Answers is that Google Answers only allowed for one answerer to provide the official answer; however, multiple answers were allowed in Mahalo and the reward went to the best answer.

Every user had a profile page in which the following information was posted: earned points, number of questions and answers, number of best answers, tips given and received along with the ranking of the users based on mentioned features. Followers, friends and following were also showed.

#### WebMD

WebMD Answers is a health question and answer service which in contextually integrated through a health public website called WebMD. It is certified by HONCode and URAC^[[6]](#footnote-6)^ for the quality of health information it provides. Users can post their question for free and they may receive an answer from their fellow users, health experts or organisations who participate on a voluntary basis. The main stream of revenue comes from online advertising.

WebMD has a simple and transparent reputation system for answers. The following information is visible for each answerer on their profile page: total number of questions answered; number of followers; number of Helpful Answer Votes they received and all answers provided by them.

#### Quora

Quora is a question and answer website launched by two former Facebook employees in 2010 and its revenue model is not established yet. Quora allows users to ask, answer and edit questions and answers. That social element is what makes Quora different from other question and answer sites like Yahoo Answers. Quora focuses on leveraging social connections to get questions answered (Ovadia, 2011). Similar to the Yahoo Answers point system, Quora uses a credit mechanism to encourage participation. Everyone on Quora starts with 500 credits and users can earn credits when people like their answers or follow their questions, however, the unique feature of Quora is that users can specify how much credit they would like to get to answer a question and askers can pay with their credits to have their questions answered. Askers can also promote their questions by spending some credits e.g. to promote a question to 100 people, 500 credits should be spent.

Quora summarises all activates of its users on their profile page to help other users judge their peers’ reputation, including: number of questions, number of answers, number of posts, number of edits, number of followers and following and all their public questions and answers. However, the users’ credit remains confidential and the amount charged to provide an answer appears at the asking point.

Table 2 summarizes the characteristics and design features of the selected platform. It should be noted that some of the design features e.g. revenue model are the same for the whole sample taken from each platform, however, some other (e.g. respondent or financial incentive) are variant inside each platform. For example in Google Answers question are answered either by lay users or certified experts. For each record we checked whether the question in our sample was answered by an expert or by a non-expert respondent and code the data accordingly.

# Table 2: Characteristics and design feature of the sample Q&A platforms

| **Q&A Platform** | **Year** | **Revenue Model** | **Respondents** | **Design features** |
| --- | --- | --- | --- | --- |
| **AllExperts** | 1998 - Present | Advertisement | Medical expert with certification | Online reputation, Offline reputation, Non-financial incentive |
| **Answerbag** | 2003 - Present | Advertisement | Lay users | Online reputation, Non-financial incentive |
| **ChaCha** | 2006 - Present | Advertisement - based | Lay users | Financial incentive |
| **Google Answers** | 2002-2006 | Transaction-based | Experts | Online reputation,  Non-financial incentive, financial incentive |
| **Just Answer** | 2003 - Present | Transaction-based | Medical expert with certification | Online reputation,  Financial incentive |
| **Mahalo Answers** | 2008 - 2013 | Mixed (Advertisement & Transaction) | Lay users | Online reputation,  Web 2.0 Mechanism,  Non-financial incentive, Financial incentive |
| **Quora** | 2010 - Present | Not established yet | Lay users | Online reputation, Offline reputation,  Web 2.0 Mechanism,  Non-financial incentive |
| **WebMD Answers** | 2012- Present | Advertisement | Mixed users | Online reputation, offline reputation, Web 2.0 Mechanism,  Non-financial incentive |
| **Yahoo Answers** | 2005 - Present | Advertisement | Lay users | Online reputation,  Non-financial incentive |

1. The 10 categories are: (1) Arts and Entertainment, (2) Business and Money, (3) Computers, (4) Family and Home, (5) Health, (6) Reference, Education and News, (7) Relationships and Society, (8) Science, (9) Sports and Recreation, and (10) Miscellaneous [↑](#footnote-ref-1)
2. (1) Arts & Humanities, (2) Beauty & Style, (3) Business & Finance, (4) Cars & Transportation (5) Computers & Internet, (6) Consumer Electronics, (7) Dining Out, (8) Education & Reference, (9) Entertainment & Music (10) Environment, (11) Family & Relationships, (12) Food & Drink, (13) Games & Recreation, (14) Health, (15) Home & Garden, (16) Local Businesses, (17) News & Events, (18) Pets, (19) Politics & Government, (20) Pregnancy & Parenting, (21) Science & Mathematics, (22) Social Science, (23) Society & Culture, (24) Sports, (25) Travel, (26) Yahoo Products [↑](#footnote-ref-2)
3. AllExperts has been mentioned in over [60 publications](http://www.allexperts.com/central/press.htm) such as The New York Times, New York Newsday, Family, PC Magazine and Yahoo (both a "Pick of the Week" and "Incredibly Useful Site"). [↑](#footnote-ref-3)
4. (1) Health & Medical, (2) Legal & Tax, (3) Cars & Vehicles, (4) Vets & Pets, (5) Home & Appliances, (6) Computer, (7) Life & Personal [↑](#footnote-ref-4)
5. ChaCha shifted its focus from text messaging service to mobile app. It also made a mobile app in which anybody can answer a question. [↑](#footnote-ref-5)
6. URAC, an independent, non-profit organisation, is a well-known leader in promoting healthcare quality through its accreditation, education, and measurement programmes. [↑](#footnote-ref-6)
