# Supplementary material for: You Get What You Pay for on Health Care Question and Answer Platforms: Nonparticipant Observational Study
Source: J Med Internet Res. 2020 Jan 15;22(1):e13534. doi: 10.2196/13534 (PMC6996747; doi:10.2196/13534)
Supplement: Multimedia Appendix 2 [file jmir_v22i1e13534_app2.docx]

**Data collection and Rating process**

Two rounds of pilot studies were run to train the assessors and refine the quality criteria.

# First Pilot Study

The first pilot study aimed at clarifying the meaning of each quality criterion to be used in a health context. A random sample of 20 questions and answers were selected from Yahoo Answers and Just Answer. The two physicians were invited to take part in the pilot study. The meaning of each rating criterion was explained before rating to both of them. Then, they were asked to rate 10 questions and answers separately. The rating session was followed by a two-hour discussion on challenges and ambiguities of the quality criteria. Their provided ratings were checked to point out the different ratings and the coders were asked to explain the reasons for their ratings. The participants rated another 10 questions to check if the rating guidelines had become more meaningful after the discussion session. The feedback from the raters was used to refine the measures.

# Second Pilot Study

The aim of the second pilot study was to overcome the technical concerns of data collection and clarify the data collection process and ratings. A random sample of 80 questions and answers were selected from four Q&A platforms. In order to facilitate the process of rating, an online tool in a form of website was designed and launched (see Figure 3). The same physicians were rated the Q&As.


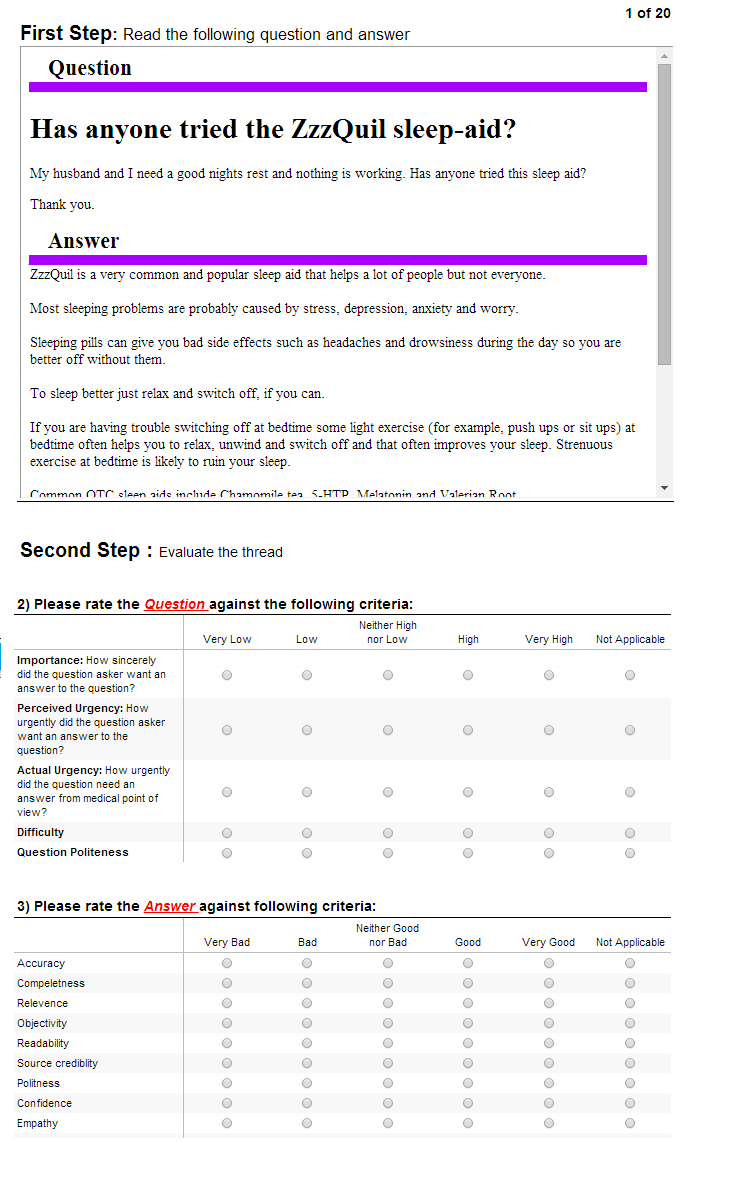


Figure 3: Snapshot of online coding tool used for second pilot study

# Main study rating

The data were collected over a period of 6 weeks from 1st of June to 15 of July 2014.

To ensure that the sample selection was random, two approaches were adopted.

(1) For the platforms which had a health category and provided a list of questions and answers raised in their platform such as Yahoo Answers, Google Answers, WebMD: 100 random numbers with a value between 1 and 1000 were generated. The questions and answers were selected in respect to generated numbers from the list of questions and answers available in their health category.

(2) For those platforms where questions and answers were accessible by a searching tool such as Quora and Just Answer, a list of 264 health-related keywords was produced to search and find health-related questions and answers. For a complete list of keywords see end of this appendix. The procedure of nominating questions and answers was as follows: a keyword was randomly selected from the health keyword list and it was searched using the search tool of the Q&A platform under study. Out of the search results, random questions and answers were chosen. For those platforms that had multiple answers for a question, one random answer was selected.

All questions were carefully reviewed and questions related to health insurance or a healthcare system such as: ‘why insurance cover Viagra not birth control pills?’ or ‘Is average life expectancy the best way to measure effectiveness of a given country’s healthcare system?’ were excluded, because different expertise is needed to evaluate the answers to these questions.

Two sets of information were needed for each Q&A thread: (1) quality rating and (2) design feature associated with Q&A (see Figure 4 ). Quality ratings were completed by human assessors and design features were assessed from the Internet presence of the platform.

After reviewing the questions and answers, they were fed into webpages and integrated with Survey Qualtrics to make an online tool for coding. The questions and answers were classified into groups of 20 each. The assessors were trained to be able to use the tool for coding efficiently in a one to one session. The questions and answers were sent to the assessors over a period of two and half months to be rated. The raters were blinded from the name of the Q&A website where the question was asked or any other attributes associated to the questions and answers, in order to ensure that information was graded independently of any site specific bias. Ten percent of the data were rated by both participants to be able to measure inter-rater consistency. Coding and data collection were completed over a three-month period from August 2014 to October 2014.

When data on both design features and quality ratings were collected the two data sets were merged. A random sample of the collected data was checked to make sure that the data had been merged correctly.

Figure 4: Data collection stages

Select a random 900 sample of Q&As

Collecting data on design features

Constructing webpages for each Q&A

Integrating webpages with Survey Qualtrics

Rating Q&As

Merging data on quality and design features

Training assessors

Health keywords

| ABDOMINAL PAIN |
| --- |
| SPINAL PAIN |
| Abortion |
| Acne |
| AIDS |
| Alcohol |
| Alcoholism |
| Allergies |
| Alternative Medicine |
| Alzheimer’s |
| Alzheimer's Disease |
| Amnesia |
| AMPUTATION |
| Anemia |
| Anesthesiology |
| Anxiety |
| Anxiety Disorders |
| ARM AND LEG PAIN |
| Arthritis |
| Artificial insemination |
| Asthma |
| Atherosclerosis |
| Athlete's Foot |
| Atopic Dermatitis |
| Attention Deficit Disorder |
| Attention Deficit Hyperactivity Disorder |
| Audiology |
| Autism |
| Avian flu |
| Back Injury |
| Back Pain |
| Bacterial |
| Bacterial Vaginosis |
| Bacterium |
| Baldness |
| Bedbug |
| Benign Prostatic Hyperplasia |
| Biology |
| Biotech |
| Bipolar Disorder |
| Blindness |
| Blood |
| BLOOD PRESSURE |
| Blood vessels |
| BODY TEMPERATURE |
| Bone grafting |
| BONE PAIN |
| Brain Tumor |
| Breast Cancer |
| BREAST PAIN |
| Breasts |
| Broken Bone |
| BURN |
| Cancer |
| Cardiology |
| Cataract |
| Celiac Disease |
| Chewing Tobacco |
| Chickenpox |
| Chlamydia |
| Chronic Disease |
| Chronic Fatigue Syndrome |
| Chronic Pain |
| Cigarettes |
| Cigars |
| Circulation |
| Cold |
| Coldness |
| Colitis |
| Colon Cancer |
| Condom |
| Congestive Heart Failure |
| Corpulence |
| COSMETIC SURGERY |
| Crohn's Disease |
| Cystic Fibrosis |
| Deafness |
| Dealing with terminal conditions |
| Death |
| Dementia |
| Dentistry |
| Depression |
| Dermatology |
| Diabetes |
| Diarrhea |
| Diet |
| Digestive |
| DILATION AND CURETTAGE |
| Disability Issues |
| Donation |
| Drowsiness |
| Drug Overdose |
| Drugs |
| Ear |
| Eating Disorders |
| Eczema |
| Emphysema |
| Epilepsy |
| Exercise |
| Fats |
| Female Ejaculation |
| Fertility |
| Fever |
| Fibromyalgia |
| First Aid |
| Flu |
| Food Allergy |
| FRACTURES |
| Gallstone |
| Gastroenteritis |
| Genital Herpes |
| Genital warts |
| Gonorrhea |
| GROIN PAIN |
| Gynecologic Cancers |
| Gynecology |
| Hair Loss |
| HAND AND FOOT PAIN |
| Hangovers |
| Hard of Hearing |
| Harmful |
| Headaches |
| Health |
| Health Care |
| Healthy |
| Heart |
| Heart Disease |
| HEART RATE |
| Heartbeat |
| Heartburn |
| Hepatitis |
| Hepatitis A/B/C |
| Hepatitis C |
| Hernia |
| Herpes |
| Herpes Simplex |
| High Blood Pressure |
| HIV |
| Hives |
| Hormones |
| Human papillomavirus |
| Hyperthyroidism |
| Hypoglycemia |
| Hysterectomy |
| Ibuprofen |
| Idiopathic Intracranial Hypertension |
| Infertility |
| Inhaler |
| Injection |
| Injuries |
| Internal organs |
| Irritable Bowel/Crohn's Disease |
| JOINT REPLACEMENTS |
| Lactose Intolerance |
| Lice |
| Liposuction |
| Low Blood Pressure |
| Lungs |
| Lupus |
| Lyme Disease |
| Malaria |
| Mammogram |
| Medical & Health Issues |
| Medical Specialists |
| Menopause |
| MENSTRUAL PAIN |
| Menstruation |
| Migraine |
| Migraine Headaches |
| Miscarriage |
| Motion sickness |
| Multiple sclerosis |
| Muscle |
| MUSCLE PAIN |
| Nausea |
| Neck Injury |
| NECK Pain |
| Nephrology |
| NERVE PAIN |
| Neuralgia |
| Neurological conditions |
| Nose |
| Nursing |
| Obamacare |
| Obesity |
| Obsessive-Compulsive Disorder |
| Obstetrics |
| Oncology |
| Organ donation |
| Organ transplants |
| Orphan Diseases |
| Orthopedics |
| Osteoarthritis |
| Osteoporosis |
| Otolaryngology |
| Paget's Disease |
| Pain |
| PAIN MANAGEMENT |
| PAINKILLERS |
| Pancreas |
| Panic |
| PAP tests |
| Parkinson's Disease |
| Pediatrics |
| Penicillin |
| Pharmacology |
| Physical Therapy |
| Pipes |
| Plastic Surgery |
| Pneumonia |
| Poisoning |
| Postpartum Depression |
| Pregnancy |
| Prostate Cancer |
| Prostatitis |
| Psoriatic Arthritis |
| Psychological |
| Public Health |
| Pulse rate |
| Quitting smoking |
| Radiation Surgery |
| Rash |
| Reaction |
| RESPIRATORY RATE |
| Ringworm |
| Schizophrenia |
| Senior Health |
| Sexuality |
| Sexually Transmitted Diseases |
| Sexually-transmitted diseases |
| Shingles |
| Side effect |
| Sinus Infection |
| Sleep Disorders |
| Smokeless ashtrays |
| Smoking |
| Sports Medicine |
| STOMACH Pain |
| Stress Management |
| Surgery |
| Swine influenza |
| Syphilis |
| TESTICULAR PAIN |
| Throat |
| Thyroid Disease |
| TONSILLECTOMY |
| Toxic Shock Syndrome |
| Toxoplasmosis |
| Transient Ischemic Attack |
| Traumatic Brain Injury |
| Trigeminal Neuralgia |
| Tuberculosis |
| Type 1 Diabetes |
| Type 2 Diabetes |
| Ulcerative Colitis |
| Ultrasound |
| Unhealthy |
| Urinary |
| Urology |
| Uterine fibroids |
| Vaccines |
| Vaginal Issues |
| Veterinary Medicine |
| Viral infections |
| Virus |
| Wart |
| Women's Health |
