# Supplementary material for: You Get What You Pay for on Health Care Question and Answer Platforms: Nonparticipant Observational Study
Source: J Med Internet Res. 2020 Jan 15;22(1):e13534. doi: 10.2196/13534 (PMC6996747; doi:10.2196/13534)
Supplement: Multimedia Appendix 3 [file jmir_v22i1e13534_app3.docx]

# Principal Component Analysis for answer quality variables

| **Communalities** | | |
| --- | --- | --- |
|  | Initial | Extraction |
| Accuracy | 1.000 | .913 |
| Relevance | 1.000 | .889 |
| Completeness | 1.000 | .850 |
| Objectivity | 1.000 | .904 |
| Readability | 1.000 | .847 |
| Source Credibility | 1.000 | .730 |
| Extraction Method: Principal Component Analysis. | | |

| **Total Variance Explained** | | | | | | |
| --- | --- | --- | --- | --- | --- | --- |
| Component | Initial Eigenvalues | | | Extraction Sums of Squared Loadings | | |
|  | Total | % of Variance | Cumulative % | Total | % of Variance | Cumulative % |
| 1 | 5.133 | 85.553 | 85.553 | 5.133 | 85.553 | 85.553 |
| 2 | .355 | 5.909 | 91.462 |  |  |  |
| 3 | .222 | 3.695 | 95.157 |  |  |  |
| 4 | .114 | 1.902 | 97.059 |  |  |  |
| 5 | .102 | 1.707 | 98.766 |  |  |  |
| 6 | .074 | 1.234 | 100.000 |  |  |  |
| Extraction Method: Principal Component Analysis. | | | | | | |

| **Component Matrix^a^** | |
| --- | --- |
|  | Component |
|  | 1 |
| Accuracy | .955 |
| Relevance | .943 |
| Completeness | .922 |
| Objectivity | .951 |
| Readability | .920 |
| Source Credibility | .854 |
| Extraction Method: Principal Component Analysis. | |
| a. 1 components extracted. | |

# Principal Component Analysis for question quality variables

| **Communalities** | | |
| --- | --- | --- |
|  | Initial | Extraction |
| Importance | 1.000 | .840 |
| Perceived Urgency | 1.000 | .542 |
| Difficulty | 1.000 | .789 |
| Question archival value | 1.000 | .837 |
| Writing quality | 1.000 | .485 |
| Extraction Method: Principal Component Analysis. | | |

| **Total Variance Explained** | | | | | | |
| --- | --- | --- | --- | --- | --- | --- |
| Component | Initial Eigenvalues | | | Extraction Sums of Squared Loadings | | |
|  | Total | % of Variance | Cumulative % | Total | % of Variance | Cumulative % |
| 1 | 3.494 | 69.870 | 69.870 | 3.494 | 69.870 | 69.870 |
| 2 | .661 | 13.214 | 83.084 |  |  |  |
| 3 | .459 | 9.183 | 92.267 |  |  |  |
| 4 | .240 | 4.807 | 97.074 |  |  |  |
| 5 | .146 | 2.926 | 100.000 |  |  |  |
| Extraction Method: Principal Component Analysis. | | | | | | |

| **Component Matrix^a^** | |
| --- | --- |
|  | Component |
|  | 1 |
| Importance | .916 |
| Perceived Urgency | .736 |
| Difficulty | .888 |
| Question archival value | .915 |
| Writing quality | .697 |
| Extraction Method: Principal Component Analysis. | |
| a. 1 components extracted. | |
