# Supplementary material for: You Get What You Pay for on Health Care Question and Answer Platforms: Nonparticipant Observational Study
Source: J Med Internet Res. 2020 Jan 15;22(1):e13534. doi: 10.2196/13534 (PMC6996747; doi:10.2196/13534)
Supplement: Multimedia Appendix 4 [file jmir_v22i1e13534_app4.docx]

# Analytical methods

## LASSO

Least Absolute Shrinkage and Selection Operator (LASSO) is a regularized regression technique with improved prediction accuracy and interpretability comparing with the popular OLS regression [43]. Similar to OLS, LASSO searches for coefficient estimates that minimize the residual sum of squares, but it forces the coefficient estimates of predictors that are unimportant or create overfitting towards zero. In other words, LASSO fits a linear model to the data, but adds a penalty term that reduces non-significant coefficient estimates to zero and thus excludes them from estimation.

Formally, the LASSO coefficients, βˆL , minimizes the following quantity:

(1)

Or similarly expressed in the form of Lagrangian:

(2)

The term λ Pp|βij | is the LASSO penalty term, also referred to as L1 penalty term. The penalty term is the L1 norm of the coefficient vector β, ||β||.

j=1

The method fits a model containing p predictors using a technique that regularizes or shrinks coefficient estimates of [insignificant] predictors towards zero. LASSO proceeds by fitting a linear model similar to the OLS, with the difference that it adds a penalty term that shrinks coefficient estimates to zero and thus excluding them from estimation.

λ

LASSO is fitted using cyclical coordinate descent algorithm which successively optimizes the objective function in (2) over each parameter with others fixed, and cycles repeatedly until convergence occur (Friedman et al., 2010). Like OLS, LASSO searches for coefficient estimates that minimize the residual sum of squares but will shrink the coefficient estimates towards zero for those predictors that are unimportant or create overfitting in the data. λ serves as the tuning parameter where λ = 0 corresponds to the full OLS model and the strength of the regularization increases as λ → ∞. The optimal value of λ is chosen using cross validation procedures. Because of the L1 penalty term, LASSO shrinks coefficient estimates that are exactly zero thereby performing feature selection. (Tibshirani, 1996) and Hastie et al.(2011) provide further details on the technique.

We employ LASSO to identify predictively significant variables and narrow down the list of predictors in our sample. The glmnet package of R software has been used to run the anlaysis.

Unbiased regression tree

The regression tree mechanism involves recursively partitioning the predictor space into a number of small regions based on simple rules and using the mean or median of the realised values of observations (e.g., quality score) belonging to a region as the predicted value for a new observation that falls in that particular region. All the splitting decision rules, order of important predictors and their interactions are summarised in a visually attractive and intuitive way. The segmentation patterns showing up in the tree help identify potential complementarities among independent variables, shedding light on how various drivers interact to shape dependent variable (information quality in our case).

In this paper, we opt to use a conditional inference framework proposed in Hothorn et al. (2006). The algorithm works as follows:

1) Test the global null hypothesis of independence between any of the input variables and the response. Stop if this hypothesis cannot be rejected. Otherwise select the input variable with strongest association to the response. This association is measured by a p-value corresponding to a test for the partial null hypothesis of a single input variable and the response.

2) Implement a binary split in the selected input variable.

3) Recursively repeat steps 1 and 2 (r help).

The technique rectifies the problem of selection bias by choosing predictors for splitting based on a series of tests identifying statistically significant associations between the responses and predictors.

As any non–parametric estimator, regression trees are subject to over-fitting. To achieve an optimal trade-off between the bias and variance (over-fitting), we rely on the out of sample predictive accuracy of the tree models, estimated using cross validation. We select the regression tree model with the lowest prediction error to identify patterns among the variables that best fit the data.
